# Microbial Keratitis Profile at a University Hospital in Hong Kong

**DOI:** 10.1155/2014/689742

**Published:** 2014-11-11

**Authors:** Tracy H. T. Lai, Vishal Jhanji, Alvin L. Young

**Affiliations:** Department of Ophthalmology & Visual Sciences, The Chinese University of Hong Kong, and Prince of Wales Hospital and Alice Ho Miu Ling Nethersole Hospital, Shatin, Hong Kong

## Abstract

*Purpose*. To evaluate the recent trends in demographics, risk factors, and microbiological profiles of microbial keratitis at a university hospital in Hong Kong. *Design*. Retrospective review. *Methods*. The medical records of 51 patients admitted to the Prince of Wales Hospital for microbial keratitis from January 2010 to June 2012 were reviewed. Demographics, risk factors, clinical features, microbiological results, and treatment were recorded. Data was analyzed and compared to our historical sampled data collected 11 years ago. *Results*. The mean age of patients was 41.6 ± 20.3 years. Contact lens use was the major risk factor (45%), followed by injury (12%). The culture positive rate was 59%, of which 37% were Gram-positive organisms and 53% were Gram-negative organisms. *Pseudomonas aeruginosa* (50%) and coagulase-negative *Staphylococcus* (13%) were the most commonly isolated pathogens. No resistance to fluoroquinolones was identified. *Conclusions*. Our study showed that contact lens wear remained the major risk factor for microbial keratitis in Hong Kong and *Pseudomonas aeruginosa* remained the commonest bacterium isolated. This is comparable to our historical data and other studies conducted in East Asia.

## 1. Introduction

Microbial keratitis is a serious condition that could result in corneal scar, corneal perforation, and even blindness. Microbial keratitis usually occurs in the presence of predisposing factors, such as ocular trauma, ocular surface diseases, and contact lens wear. The demographics and microbiological profile of microbial keratitis vary across countries and different studies have been published across the world [1]. Major studies have been conducted in Hyderabad, India [2], Miami, USA [3], and Oxford, UK [4]. Shifting trends in the microbiological profile of keratitis have been reported in studies in some parts of the world [5, 6]. Therefore, it is important to carry out studies periodically to review local organisms and sensitivities. For instance, increasing resistance to fluoroquinolones has been reported in a study in Florida [3] and a recent study in Toronto found a decreasing trend in the percentage of Gram-positive bacteria in the past 11 years [6]. The last Hong Kong-based study on incidence and risk factors for microbial keratitis was reported in 2002 [7]. The purpose of the current study is to examine the demographics, risk factors, microbiological results, and treatment given for adult microbial keratitis patients requiring admission to a university hospital in Hong Kong from January 2010 to June 2012.

## 2. Materials and Methods

This is a retrospective review conducted for a single centre to isolate cases with presumed infective microbial keratitis. Patients were identified using the electronic record system and patients who were admitted between January 2010 and June 2012 with a discharge diagnosis matching either corneal ulcer (ICD-9 code: 370.00) or corneal disorder due to contact lens (ICD-9 code: 371.82) were identified. Outpatients were excluded from the current study.

Fifty-one medical records were tracked and data was retrieved. Microbial keratitis was defined by the presence of a corneal infiltrate >1 mm^2^ in size with or without overlying epithelial defect [7]. Corneal scraping was performed under topical anesthesia following a standard protocol. Corneal specimens were collected using Kimura spatulas. Specimens were placed on glass plates for Gram Stain and also on blood agar, chocolate agar, Sabouraud agar, and thioglycollate broth. In cases that were nonresponsive to treatment and clinically suspicious of* Acanthamoeba*, culture would then be taken for* Escherichia coli* plates for* Acanthamoeba*.

The sex, age, risk factors, corneal scraping culture results and sensitivity profiles, and antibiotics prescribed were recorded and analyzed. Institutional Review Board/Ethics Committee's approval was not required for this study. The study adhered to the tenets of the Declaration of Helsinki.

## 3. Results

Among the 51 patients, 29 (57%) were male and 22 (43%) were female. The mean age was 41.6 ± 20.3 years (range 20 to 86 years). Regarding the risk factors, 23 patients (45%) were contact lens wearers, 6 (12%) had corneal foreign body, 5 (10%) had recurrent corneal erosion, and 4 (8%) had corneal graft (Table 1). The cases with corneal erosions and bullous keratopathy were not related to contact lens use.

Among the 23 contact lens wearers, 7 (30%) used monthly disposable contact lens, 10 (43%) used 2-week disposable lens, one (4%) used daily disposable lens, and two (7%) used colored contact lens. Three patients practiced overnight wear, one patient reused a daily disposable contact lens, and one patient cleansed the contact lens every 2-3 days. The mean age for contact lens wearers was 27.7 ± 6.7 years, which was significantly lower than the mean age of non-contact lens wearers (*P* < 0.05). Interestingly, none of the cases in our study were related to* Acanthamoeba*.

Thirty (59%) out of the 51 patients had positive corneal scraping results. Gram-positive organisms were cultured in 17 eyes and Gram-negative organisms were cultured in 11 eyes. Two patients had polymicrobial growth.* Pseudomonas aeruginosa* was the most prevalent pathogen (50%). Out of the 15 patients who had* Pseudomonas*, 13 were contact lens wearers. The other pathogens were coagulase-negative* Staphylococcus* (4 patients, 13%) and* Staphylococcus aureus* (2 patients, 7%). Out of the* Pseudomonas* ulcers with sensitivity profile done, all were sensitive to gentamicin and ciprofloxacin. One sample showed intermediate sensitivity to ticarcillin and clavulanate but no antibiotic resistance was identified. Out of the 23 contact lens related ulcers,* Pseudomonas aeruginosa* was the pathogen in 13 cases (57%).  Table 2 shows the relationship between risk factor and microbiological profile.

Only one patient was given monotherapy upon admission, while the rest were given combined therapy. Thirty-three patients were prescribed ceftazidime + tobramycin, 11 patients were prescribed vancomycin + tobramycin, and two patients were prescribed vancomycin + ceftazidime, while three patients had acyclovir ointment in addition to fortified antibiotics. Two culture negative patients were given natamycin and amphotericin, respectively, as fungal keratitis was suspected.

The average logMAR visual acuity on presentation was 0.99. Visual acuities of hand motion and counting finger were assigned a decimal visual acuity of 0.005 and 0.014 according to the Freiburg Visual Acuity test [8]. Visual acuity of light perception or no light perception could not be quantified and was excluded from the calculation of the average visual acuity. At one month and three months after presentation, the average logMAR visual acuity improved to 0.34 and 0.26, respectively. Due to patients defaulting follow-up or being discharged from clinic, visual acuities of only 37 and 19 patients were documented at one month and three months, respectively. Among the 19 patients, the improvement in average logMAR visual acuity was −0.77 at three months.

## 4. Discussion

### 4.1. Predisposing Factors

In our study, contact lens wear was the most important risk factor, accounting for 45% of all cases with microbial keratitis. Contact lens wear is the major risk factor for microbial keratitis in developed countries. For example, contact lens wear accounted for 34% and 50% of microbial keratitis in studies in Australia [9] and France [10], respectively. In contrast, trauma was the major risk factor for microbial keratitis in developing countries. Trauma accounted for 48%, 53%, and 83% of the microbial keratitis cases in Paraguay [11], South India [12], and Eastern India [13], respectively. Patients often had injury during farming and higher rates of injury were reported during the harvesting season [12].

In our study, 3 (13%) out of the 23 contact lens wearers with microbial keratitis reported overnight wearing of lenses. In a study by Lam et al. [7] in 2002, overnight wear was identified as a significant risk factor for microbial keratitis (*P* < 0.0001). In a study conducted by Yildiz et al. [14], 54% of contact lens wearers with microbial keratitis reported history of overnight wear.

In contrast to our previous studies, none of the patients used orthokeratology lens for the current review period [15, 16]. This may be a result of the enhanced public awareness and education on the risks following our previous media reports and publications. However, in a recent Hong Kong series of pediatric ocular surface infections, 9 (7%) out of 138 patients used orthokeratology lens [17].

### 4.2. Microbiology

In our study, 59% of corneal scrapings yielded positive cultures. This rate may be related to the fact that patients were often given topical antibiotics by general practitioners or at the Accident and Emergency Department prior to presentation in our clinic. However, this figure is comparable to other major studies. Studies in Germany [18], Australia [9], Texas [19], and Oxford [4] yielded culture positive rates of 43%, 49%, 53%, and 54%, respectively.

The microbiological profile of microbial keratitis varies across countries. In our study,* Pseudomonas aeruginosa* was the most common pathogen, similar to a study conducted in Hong Kong 11 years ago [7]. This is similar to other studies conducted in East Asia, in mostly “urban” population. According to two studies in Taipei [20, 21], two studies in Singapore [22, 23], and one study in Bangkok [24],* Pseudomonas aeruginosa* was the most prevalent pathogen, accounting for 29%–42% of microbial keratitis cases. This was different from studies conducted in Australasia [9], North America [3], Canada [25], and Europe [26], where staphylococci were the most common bacteria. In particular, for three studies conducted in France [10], Switzerland [26], and Turkey [27], the prevalence of staphylococciwas as high as 52 to 60%.

There was no documented case of fungal infection in our series. This was very different from studies conducted in more rural areas, where more people practiced agriculture and therefore fungal infection was much more prevalent. In Eastern India, fungal infections accounted for 67% of microbial keratitis cases [13].* Aspergillus* accounted for 60% of fungal cultures in Eastern India [13] and* Fusarium* accounted for 73% of fungal cultures in a study in Hyderabad, India [2]. The absence of fungal keratitis in our series might be attributed to the fact that majority of the patients came from an urban background and did not have a history of trauma with vegetative material as is classically seen in cases with fungal keratitis. Furthermore,* Acanthamoeba* keratitis is not very commonly seen in our setting nowadays possibly due to an enhanced level of knowledge regarding contact lens care amongst the general population in Hong Kong. Also, majority of* Acanthamoeba* keratitis cases are being treated on an outpatient basis in our setting.

Resistance to erythromycin, clindamycin, cloxacillin, and cotrimoxazole was reported in four samples. These antibiotics are seldom used in the treatment of microbial keratitis in our centre. No resistance to fluoroquinolones was demonstrated in our study, in contrast to reports from the United States [3] and India [28, 29], which showed persistent resistance to fluoroquinolones.

There are several limitations of the present study. As this is a retrospective study, clinical data such as the exact percentage of patients who received antibiotic treatment before presentation were not available. This would surely contribute to a lower percentage of positive scraping results. Some of the suspected cases of microbial keratitis might have been sterile contact lens related inflammatory infiltrates. In addition, the sizes of infiltrate were not documented in all cases and thus it was not possible for a more detailed analysis. Moreover, patients with less severe microbial keratitis managed in the outpatient eye clinic were not included in this study, thus limiting the sample size of our study.

## 5. Conclusions

In summary, our study showed that contact lens wear remained the major risk factor for microbial keratitis in Hong Kong and* Pseudomonas aeruginosa* is the commonest bacterium isolated. This is comparable to a study conducted in Hong Kong 11 years ago and other studies conducted in East Asia.

## Figures and Tables

**Table 1:** Demographic data of patients with microbial keratitis treated at a university hospital in Hong Kong between 2012 and 2014.

	Number of patients	%
Age (years)		
<20	3	6
21–39	25	49
40–64	14	27
>65	9	18
Sex		
Male	29	57
Female	22	43
Risk factors		
Contact lens wear	23	45
Foreign body	6	12
Recurrent corneal erosion	5	10
Corneal graft	4	8
Skin condition	2	4
Exposure keratopathy	2	4
Bullous keratopathy	1	2
Herpetic keratitis	1	2
Neurotrophic ulcer	1	2
Ocular trauma	1	2
Trichiasis	1	2
No risk factor	4	8

**Table 2:** Organisms isolated and predisposing factors in patients with microbial keratitis cases treated at a university hospital in Hong Kong between 2012 and 2014.

Microbiological result/risk factor	CL	Foreign body	RCES	Corneal graft	Skin condition	Exposure keratopathy	Others
Gram-positive bacteria							
Coagulase-negative *Staphylococcus *			2			1	1
*Staphylococcus aureus *		1					1
Other *Streptococcus *	1						
*Streptococcus pneumoniae *		1		1			
Diphtheroids		1					1
Gram-negative bacteria							
*Pseudomonas aeruginosa *	13	1		1			
*Citrobacter *				1			
Mixed isolates						1	1
Negative culture result	9	2	3	1	2		5

Total	23	6	5	4	2	2	9

CL: contact lens; RCES: recurrent corneal erosion syndrome.
